# Nutrition Risk, Resilience and Effects of a Brief Education Intervention among Community-Dwelling Older Adults during the COVID-19 Pandemic in Alberta, Canada

**DOI:** 10.3390/nu14051110

**Published:** 2022-03-06

**Authors:** Michelle Capicio, Simran Panesar, Heather Keller, Leah Gramlich, Naomi Popeski, Carlota Basualdo-Hammond, Marlis Atkins, Catherine B. Chan

**Affiliations:** 1 4-126 Li Ka Shing Centre of Health Innovation, Department of Agriculture, Food and Nutrition Science, University of Alberta, Edmonton, AB T6G 2E1, Canada; mcapicio@ualberta.ca; 2 Department of Physiology, University of Alberta, 7-55 Medical Sciences Building, Edmonton, AB T6G 2H7, Canada; spanesar@ualberta.ca; 3 Department of Kinesiology and Health Sciences, and Schlegel-UW Research Institute for Aging, University of Waterloo, Waterloo, ON N2G 0E2, Canada; hkeller@uwaterloo.ca; 4 Department of Medicine, Division of Gastroenterology, University of Alberta, Edmonton, AB T6G 2G3, Canada; lg3@ualberta.ca; 5 Diabetes, Obesity and Nutrition Strategic Clinical Network, Alberta Health Services, Calgary, AB T2W 1S7, Canada; npopeski@ucalgary.ca; 6 Department of Community Health Sciences, Cumming School of Medicine, University of Calgary, Calgary, AB T2N 1N4, Canada; 7 Nutrition Services, Alberta Health Services, Edmonton, AB T5J 3E4, Canada; carlota.basualdo@ahs.ca (C.B.-H.); marlis.atkins@ahs.ca (M.A.)

**Keywords:** coronavirus, nutrition risk, resilience, food security, aging, community dwelling, self-isolation, quarantine, malnutrition, frailty

## Abstract

Up to two-thirds of older Canadian adults have high nutrition risk, which predisposes them to frailty, hospitalization and death. The aim of this study was to examine the effect of a brief education intervention on nutrition risk and use of adaptive strategies to promote dietary resilience among community-dwelling older adults living in Alberta, Canada, during the COVID-19 pandemic. The study design was a single-arm intervention trial with pre–post evaluation. Participants (N = 28, age 65+ years) in the study completed a survey online or via telephone. Questions included the Brief Resilience Scale (BRS), SCREEN-14, a brief poverty screen, and a World Health Organization-guided questionnaire regarding awareness and use of nutrition-related services and resources (S and R). A brief educational intervention involved raising participant awareness of available nutrition S and R. Education was offered via email or postal mail with follow-up surveys administered 3 months later. Baseline and follow-up nutrition risk scores, S and R awareness and use were compared using paired *t*-test. Three-quarters of participants had a high nutrition risk, but very few reported experiencing financial strain or food insecurity. Those at high nutrition risk were more likely to report eating alone, compared to those who scored as low risk. There was a significant increase in awareness of 20 S and R as a result of the educational intervention, but no change in use. The study shows increasing individual knowledge about services and resources in the community is not sufficient to change use of these services or improve nutrition risk.

## 1. Introduction

Nutrition risk occurs in up to 70% of Canadian community-dwelling older adults [1] and is associated with hospitalization, reduced health-related quality of life, and mortality [2,3]. Nutrition risk in older adults living in the community setting is defined as the presence of risk factors that can impair food intake, and if unchecked, can result in malnutrition [4,5]. Common barriers to adequate food intake among older adults include reduced physical function, having multiple medical conditions, social isolation, financial constraint, chewing disabilities, and limited access to social supports [6,7,8]. Identifying and reducing nutrition risk in this population is important because malnutrition is associated with frailty, characterized by decreased functional capacity and increased vulnerability to environmental stressors [9,10]. Interventions to reduce nutrition risk in community-dwelling older adults might prevent malnutrition. Written materials such as handouts or brochures are acceptable to older adults [11] and can increase nutrition knowledge, although they appear to be less efficacious for changing eating habits [11,12].

The novel coronavirus disease (COVID-19) is a new, additional stressor, which has led to lockdown mandates that may contribute to further functional decline in older adults [13,14,15,16]. Poor adaptability is exacerbated by age-related changes in the immune system, such as chronic, low-grade inflammation and immunosenescence [17]. Nutritional status is a modulator of immune function outcomes related to COVID-19 [18]. This is significant because COVID-19-related calls to the Alberta 211 Helpline showed food was one of the top, unmet basic needs of Albertans during the lockdown beginning in March 2020 [19]. Studies from Europe indicate some improvements in dietary habits (e.g., higher Mediterranean diet score) during lockdown [20,21,22,23] coupled paradoxically with weight gain, perhaps due in part to enforced physical inactivity and increased snacking [20,24] but these studies had few older adults and did not stratify the results by age. The NutriQuébec study conducted in Canada found a 1.1-point increase in the Healthy Eating Index-2015 in adults, comparing April–May 2020 to June 2019–March 2020. However, adults ≥ 70 years experienced an average decrease of 0.6 points [25] suggesting the potential for increased nutrition risk.

The aging trajectories for those ≥65 years are heterogenous, with some older adults experiencing accelerated aging and more frailty [26]; this suggests that some older adults may be more resilient and better manage their health than others. Neurobiological factors and psychosocial characteristics of resilience have been defined [26,27], but do not focus on the older adult or their nutrition. A concept of dietary resilience has been described as being able to implement adaptive strategies despite challenges and stressors [28]. One theme of dietary resilience is “getting help when you need it”, meaning a willingness and ability to reach out to formal or informal supports to continue to eat well. Formal supports refer to community organizations, services from senior residences, and grocery stores, while informal supports include family members, friends, and neighbors [28]. When these services are used, the positive effect is dietary resiliency, or a high-quality, adequate diet [28].

Little is known about what nutrition-related coping strategies are used by community-dwelling older adults and how they contribute to enhanced dietary resilience. An older adult’s coping strategies are enhanced when they believe strategies to be both efficacious (able to resolve the problem) and easily implemented [29]. Understanding the adaptive strategies that older adults find useful may offer insights for interventions to increase their dietary resilience and nutritional status. Furthermore, researchers, healthcare professionals and community organizations face challenges in how to support this vulnerable population during the pandemic [16]. Thus, we aimed to examine the effect of an education intervention on nutrition risk and dietary resilience among community-dwelling older adults living in Alberta, Canada during the COVID-19 pandemic. Specifically, the objectives were to (1) measure nutrition risk, general resilience and dietary resilience (i.e., the use of food and nutrition-related supportive services) in older adults during the COVID-19 pandemic in 2020, and (2) determine if there was a change in nutrition risk, awareness and use of local formal supports related to transportation or food delivery, telephone- or internet-based health advice, nutrition counselling or food security agencies after providing the intervention (aka dietary resilience). It was hypothesized that the intervention would improve these outcomes.

## 2. Materials and Methods

### 2.1. Study Population

Participants were a convenience sample recruited through partnerships with community organizations in Alberta, including the Westend Seniors Activity Centre and Alberta Diabetes Institute in Edmonton, the Golden Circle in Red Deer, and Alberta 211 Helpline. Flyers provided telephone or email information for participants to contact a research assistant to complete a survey. Eligibility was based on the following inclusion criteria: ≥65 years old, community-dwelling and either living alone or with a spouse/roommate, and currently or previously isolating because of COVID-19. Those who reported living with their children were excluded from the study. The study was approved by the University of Alberta Research Ethics Board (Pro00100851) and participants provided verbal consent via telephone or implied consent via completion of the internet-based survey. Specifically, internet participants received an instruction that submitting the survey indicated their consent.

### 2.2. Study Design

The design is a 3-month, single-arm, pre–post intervention. Participants completed a baseline survey, after which they immediately received an education intervention developed by Alberta Health Services (AHS) (see Intervention) as well as information about available nutrition-related services and resources (S and R, including transportation or food delivery, telephone- or internet-based health advice, nutrition counselling or food security agencies). Twenty S and R were selected based on their availability in Alberta as well as their ability to address the diverse aspects of nutrition risk. Participants repeated the same survey 3 months later. Surveys were completed independently online via Research Electronic Data Capture (REDCap) or by telephone with a research assistant (MC). If requested, participants completing the surveys by telephone were provided clarification, for example of what constitutes protein foods. For SCREEN-14, prompts followed suggestions from the SCREEN interviewing guide. Based on pretesting, some prompts were also added to the online survey. Baseline surveys began in July 2020, but participants were asked to consider their experiences during the period beginning in March 2020 when isolation at home was recommended by the Government of Alberta. Follow up surveys were completed by December 2020.

### 2.3. Outcomes

The survey collected information regarding nutrition risk, general resilience, awareness and use of formal supports (i.e., dietary resilience), and self-reported poverty and food insecurity. Resilience was measured using the 6-item Brief Resilience Scale (BRS) [29], which was recommended by the World Health Organization (WHO) when studying behavior during COVID-19 [30]. A score < 3.6 out of 5 is considered low resilience [29]. Participants were screened for nutrition risk using the 14-item Seniors in the Community: Risk Evaluation for Eating and Nutrition (SCREEN-14), with a score ≤ 50 considered high risk [31]. Participants were screened for financial strain and food insecurity using 2 single-item (yes/no) questions extracted from a 9-item survey developed by Brcic et al. [32]. Poverty was reported with the question “Do you have difficulty making ends meet at the end of the month?”, and food insecurity was determined with “In the past year, was there any day when you or anyone in your family went hungry because you did not have enough money for food?” Using a WHO-guided questionnaire [30], participants were asked about awareness (yes/no) and frequency of use (never/rarely/sometimes/often/always) of 20 formal S and R relevant to food acquisition and health/nutrition advice available in the participant’s local community. Use of these services was considered an adaptive strategy to promote dietary resilience. Dietary resiliency itself was not measured as this would have required an estimation of the adequacy and quality of the participant’s diet [28]. Whether the participant read the educational materials before the post-intervention survey was not assessed.

Baseline demographic data were collected, including age, gender, living situation (alone or with spouse/roommate), self-isolation status, quarantine status, and estimated annual income. In the survey, “self-isolation” was defined as separation from other people to prevent COVID-19 from spreading. “Quarantine” was defined as separation from other people following exposure to COVID-19 or having been in close contact with someone with diagnosed COVID-19 and included monitoring one’s progression to becoming ill.

### 2.4. Intervention

Participants were provided a package that consisted of 2 educational handouts developed by AHS (www.ahs.ca. Accessed on 10 February 2022). These handouts were developed using plain language and health literacy principles. The package was either sent via Canada Post or emailed to participants, according to their preference, immediately after completion of the baseline survey. The first handout is entitled Stay Strong with Nutrition: Seniors and COVID-19 [33]. This handout shares strategies that older adults could employ to obtain adequate food intake and maintain immune health. It also includes information about the importance of staying active, and the webpage URLs and contact information for several community S and R. The second handout is entitled COVID-19: Nutrition for Recovery [34] and offers nutrition-related strategies participants could employ if they acquired a COVID-19 infection. Other S and R information was obtained from Alberta 211 Helpline (https://ab.211.ca. Accessed on 10 February 2022) and A Guide to Mobility and Independence [35].

### 2.5. Statistical Analyses

Demographic characteristics were analyzed using descriptive statistics (mean ± SD for continuous variables and % for categorical variables) for the entire sample and separately for men and women. Unpaired *t*-tests were used to compare continuous variables and Fisher’s Exact Test for categorical variables between men and women. SCREEN-14 and BRS were scored according to respective survey guidelines. Awareness and use of S and R were summed to produce a composite score, and the proportion of participants was tallied for each. Because usage was low, frequency was not analyzed. Paired *t*-tests were conducted to compare continuous pre–post intervention variables. Fisher’s Exact Test was used to compare the proportions aware of or using S and R. Differences in the SCREEN-14 between low- and high-risk individuals was compared by unpaired *t*-test. The relationship between resilience and nutrition risk was explored using Pearson correlation. Data were analyzed with GraphPad Prism version 9.3.0. *p*-values < 0.05 were considered statistically significant and <0.1 were noted as trends.

## 3. Results

### 3.1. Baseline Characteristics

A total of 29 people consented to participate, with 17 (59%) choosing the online survey. One participant was unable to be contacted for follow-up. Table 1 shows the baseline characteristics of n = 28, stratified by gender (men = 13; women = 15). Participants averaged 73 years, with 43% living alone. Gender differences were observed for self-isolating behavior (women > men, *p* = 0.07), income (men > women, *p* = 0.08) and resilience (men > women, *p* = 0.06). Nutrition risk was similar between men and women and low proportions of the participants screened positive for financial strain or food insecurity.

### 3.2. Effect of Intervention on Resilience, Nutrition Risk, S and R Awareness and Use

Table 2 shows the lack of change in nutrition risk scores at baseline versus follow-up. We found a significant positive correlation between baseline BRS and SCREEN-14 scores (r = 0.60, *p* = 0.0008).

Overall, the mean number of 20 S and R that participants reported awareness of at baseline was 10 (Table 2). The majority of participants reported awareness of restaurant and fast-food delivery, online grocery shopping and/or delivery, Meals on Wheels^TM^, Health Link 811, and food banks/food hampers. Meanwhile they were least aware of Bag-Half-Full^TM^, the AHS Rehabilitation Advice Line, and several websites with food-related resources. The intervention elicited a small but significant increase in awareness to a mean of 11 services and resources (*p* = 0.014). At baseline, use of any of the services and resources was low with even the most used services/resources accessed by less than half of the participants (maximum 43%). Use did not change after the intervention.

Awareness of transportation and food delivery services was moderate (39%) and increased significantly (*p* = 0.014) after the intervention. However, this did not translate into increased use of such services. Awareness of telephone-based health-related services was highest of all the categories at baseline (55%) and did not increase post-intervention, nor did use. Knowledge of nutrition counselling services was moderate at baseline (46%); neither awareness nor usage was significantly increased by the intervention. However, it was noteworthy that use of both AHS and primary care network counselling services doubled after the intervention even though participants were not told of their baseline nutrition risk score. The participants were generally aware of food security resources such as food banks and community organizations providing low-cost meals (50%) but <10% of them utilized these agencies.

To determine if the resources provided had actually addressed the nutrition risk factors queried in SCREEN-14, information in the AHS and S and R handouts was compared with individual risk factors (Table 2). The educational materials did not address eating alone or avoiding specific foods but did cover the other risk factors.

### 3.3. Nutrition Risk Behaviors

A score of <50 on the SCREEN-14 questionnaire indicates the participant is at high nutrition risk, i.e., engages in behaviors or experiences that could potentially lead to malnutrition. Individual items with scores ≤ 2 aid in identifying specific factors contributing to overall risk. Scrutiny of individual SCREEN-14 questions identified the specific behavior or factors contributing to nutrition risk (Table 3) for low- and high-risk individuals. Post-intervention data were used because there was no difference in pre–post nutrition risk scores. Both high- and low-risk groups scored ≤ 2 for weight perception. However, high-risk participants also scored ≤ 2 in vegetable and fruit intake, protein intake, milk and soy intake; these scores were also significantly lower than for the low-risk group. The high-risk group also had scores ≤ 2 for eating alone and limiting or avoiding foods. Both groups scored above the risk threshold for difficulty in preparing meals and problems obtaining groceries, which may be indicators of adaptive strategies to promote dietary resilience.

## 4. Discussion

We examined the effect of an education intervention on adaptive strategies to promote dietary resilience and reduce nutrition risk among community-dwelling older adults living in Alberta, Canada during the COVID-19 pandemic. The intervention included nutrition education focused on eating well to promote immunity during the pandemic and recovering from COVID-19 plus information about 20 S and R related to food. There is limited evidence regarding the type of supports and strategies older adults find useful in maintaining nutrition status, particularly during the COVID-19 pandemic. In a small convenience sample, we found normal resilience as measured with the BRS despite most people (21 out of 28) having high nutrition risk. However, high BRS scores (more resilience) were associated with a higher SCREEN score and thus a lower likelihood of nutrition risk. Knowledge of several services and resources was moderate–high but the most utilized included restaurant or fast-food delivery (29%), medical advice via Alberta Health Link 811 telephone line (28%) and MyHealth Alberta web portal (39%). The educational intervention did not change nutrition risk or use of services, although knowledge of services improved. However, we noted a doubling of the utilization of AHS and PCN dietitian counselling that, while not statistically significant, may indicate an important trend for follow-up study.

Nutrition risk is highly prevalent among community-dwelling older adults in several countries [1], and a U.S. study showed that older age increased the risk of severe COVID-19 outcomes conferred by a history of malnutrition [36], although malnutrition per se may not increase the risk of contracting COVID-19 in older people [37]. Moreover, older adults are prone to developing frailty due to vulnerability to environmental stressors [9,10] such as a pandemic with mandated lockdown strategies that reduce physical contact with others and restrictions on shopping and travel. We found that nutrition risk was similar to other studies of the Canadian population [1] and was stable over the 3 months of the study, unlike Ghanem et al. [38], who reported that malnutrition approximately doubled in prevalence during a one-month lockdown in France in March–April 2020. However, the population in that study was older, more frail and also had home care support suspended for that month, which likely exacerbated their decline in nutrition [38]. On the other hand, we did not have pre-pandemic information on the nutrition risk of our study group.

Dietary resilience, defined as being able to implement adaptive strategies despite challenges, is less well-understood than nutrition risk but focuses on common barriers to eating well in times of stress [28]. General resiliency in the study population was within the normal range and indicators of dietary resilience queried in the SCREEN-14, such as difficulty with meal preparation and problems obtaining groceries, were not different between people with low versus high nutrition risk. However, a cross-cultural study has shown approximately 24% of Canadian older adults reported having difficulty obtaining groceries, particularly in winter [1]; thus, the timing of our study may not have captured the full extent of food acquisition difficulties. Resilience scores at the low end of the normal range may indicate that many in our sample perceive difficulties in bouncing back from the pandemic, particularly women, who had lower BRS scores than men as well as lower income and a higher tendency to report self-isolating. Interestingly, lower BRS scores in women are reported by others [39]. Social isolation is a well-established challenge that this age group faced even prior to COVID-19 [40], and some participants may have developed enabling adaptations. Financial hardship, which contributes to poor resilience during COVID-19 [41] and is associated with food insecurity, was not reported by most participants. Only one participant screened positive to the food insecurity question, which asked about hunger. In Canada, older adults are less likely to experience food insecurity due to their Guaranteed Annual Income [42]. Most people age ≥65 years receive government pensions, which likely sheltered them from catastrophic loss of income. Even though the income of women in our study was about half that of men, there was no gender difference in nutrition risk score, perhaps because women possess higher dietary resilience strategies with respect to shopping and meal preparation.

People may benefit from supports tailored to the root cause of their nutrition risk; however, this would require individualized education. Increasing knowledge of available S and R related to nutrition, food security and general health could be a feasible strategy to improve nutrition risk in a large population; thus, we piloted a simple paper- or email-based intervention that included AHS-developed nutrition pamphlets/websites and information about not-for-profit agencies offering food-related services in Alberta. We hypothesized that the intervention would translate into increased usage of these supports, but while knowledge marginally increased, there was no change in use of S and R. However, the convenience sampling may not have captured participants who could have benefited the most. Statistics Canada shows that for Albertans aged ≥ 65 years, the average income for both men and women was CAD $40,000–50,000 for the years 2015–2019 [43], whereas many participants had incomes well above this average. Older adults may prefer to use informal over formal supports, as shown in qualitative studies that found community-dwelling older adults rely heavily on family and friends [44,45]. Future interventions should consider ways to enable social/community engagement [46] because increasing knowledge at the individual level was not sufficient to induce behavior change. Moreover, a gap in understanding optimal methods for communicating nutrition risk and information to older adults has been identified [47]. A benefit of providing handouts or brochures is that older adults find them acceptable, can tailor the information they read to their needs and re-read if necessary [11].

For some S and R, participants reported relatively high awareness but low use, including Meals on Wheels^TM^ and food banks. In addition to their financial independence, participants may not have considered using food banks due to social stigma, a major limitation of this type of community resource within high-income countries [48]. Only about 21% of food-insecure households used food banks in Canada, preferring financial assistance from family and friends as a strategy to avoid social stigma tied to using charitable services [49]. Food bank use might also increase if the nutrient density of foods generally offered by food banks could be improved [50]. The information gained in our study may be useful for S and R emerging in response to the COVID-19 pandemic. For example, Bag Half Full^TM^, a student-led initiative that launched during the pandemic, provides grocery delivery services similarly to Meals on Wheels^TM^. Given the lack of use of Meals on Wheels^TM^, attempts to increase awareness of these community initiatives may not be an efficient strategy for mitigating nutrition risk among older adults. Community organizations offering such services may need to adjust their marketing strategies to the demographic of individuals who may benefit from them. For example, social prescribing of food sources is acceptable and improves nutrition in people with food insecurity [51].

In contrast, we identified health-related S and R that participants do rely on, particularly use of the Health Link 811 telephone line and the MyHealth Alberta web portal, which are both marketed as credible sources of health-related information for Albertans. Linking these resources to the Alberta 211 Helpline might be helpful in expanding its uptake, to assist with food-related inquiries.

The nutrition risk behaviors in those who scored high vs. low risk on the SCREEN-14 included food avoidance, inadequate intake of important food groups, and eating alone, which is particularly concerning, given increased social isolation during COVID-19. Statistics Canada estimates that, pre-pandemic, 15% of Canadian older women and 10% of Canadian older men report feeling socially isolated [40]. Ghanem et al. [38] found that increased social isolation was a bigger driver than diet-related factors of the lockdown-related increase in malnutrition in older adults. Research shows a consistent link between social isolation and all-cause mortality, cardiovascular disease, and poor mental health outcomes [52]. Anorexia in aging and the psychosocial factors that contribute to it, specifically, depression and the deterioration of social networks, have been discussed as major contributors to the loss of motivation to eat among older adults [53]. Anxiety about exposure to COVID-19 may have prolonged social isolation even after relaxation of government-imposed lockdown. Moreover, programs developed to mitigate this issue in normal circumstances (e.g., community dining, senior centers) were closed during the pandemic lockdown. A weakness of the educational package provided to participants was lack of strategies or S and R that could mitigate eating alone. Although older adults exhibit resiliency, as shown here, their physical, cognitive and social frailty still increases their vulnerability [16]. Researchers, healthcare professionals and community organizations need to consider substitutions or creative alternatives to help meet the psychosocial needs of older adults when eating. Some clients may be willing to adopt internet- or smart phone-based programs, as envisaged during a recent workshop on preventing malnutrition [47].

Interestingly, we found a small upward trend in people seeking non-physician nutrition counselling resources after the education intervention. Six individuals (five at high nutrition risk) reported seeking nutritional counselling post-intervention who had not indicated this practice at baseline, either through AHS or their primary care network. That this occurred during a period of restricted access to healthcare and predominantly telephone-based consultations [54] is encouraging. However, although interventions that combine newsletters with telephone follow-up have increased benefit in nutrition knowledge, nutrition attitudes and behaviors remain unchanged [55]. Conversely, telehealth or other virtual healthcare approaches are deemed useful for managing chronic diseases such as diabetes [56] and are also useful when face-to-face meetings are impractical. Concurrent with this project, the Alberta Healthy Living Program developed an online 2 h workshop entitled “Staying Strong and Healthy as We Age”, in part to address nutrition risk and social isolation. More research on both the content and method of delivery of programs for older adults with malnutrition is required.

A strength of the study was use of the SCREEN-14, which has been validated specifically among Canadian older adults in the community setting. The primary strength of the SCREEN-14 is its ability to identify early determinants of inadequate food intake and weight change, with the primary goal of interrupting the maladaptive behaviors potentially leading to full malnutrition [31]. Other benefits include its ability to raise awareness of nutrition behaviors among participants, potential for self-administration, and multi-modal validity (i.e., in-person, telephone, or online). Alternatives may require in-person anthropometric measurements, which would not have been feasible in the COVID-19-restricted setting. Although, a limitation of the SCREEN-14 (or any survey) is that information may not be reliable if retrieved from participants experiencing cognitive or memory deficits, leading to recall bias. Additional limitations of this study include the sampling methods, small sample size, lack of race or ethnicity information and lack of diverse economic status. Volunteer and selection biases may have occurred because participants were recruited through partnerships with community organizations, raising the possibility that they were already engaging in adaptive strategies. It is possible that responses may have differed between online versus telephone respondents, but this was not assessed. Overall, it was challenging to study this potentially vulnerable population amidst a pandemic, where the majority were self-isolating for safety and it was not possible to recruit using personal contact (for example, by visiting senior centers); this needs to be taken into consideration for future work. In consideration of potential cognitive impairments among this population, the design of the intervention was deliberately kept simple to reduce the volume of information that was provided. Lack of change in SCREEN-14 scores may also be attributed to the education provided, which did not directly target all risk factors reported on SCREEN-14, in particular avoiding certain foods and eating alone.

## 5. Conclusions

During the COVID-19 pandemic, seeking to improve dietary resilience and reduce nutrition risk only through increased knowledge was insufficient to induce behavior change. However, options to reach the target population are limited during periods of lockdown. The study also suggests that older adults, despite their general resilience, may not consider using formal supports to improve nutrition status during times of stress. Overall, more research is needed to determine what strategies older adults find meaningful and useful in optimizing their nutrition status during public health emergencies. Additional avenues for future study include assessing strategies to increase usage of professional nutrition counselling by older adults as well as the potential impact of gender income gap on dietary resilience.

## Figures and Tables

**Table 1:** Demographic characteristics at baseline.

Characteristic	Men (N = 13)	Women (N = 15)	Total (N = 28)	*p*-Value (Men vs. Women)
Age, years ± SD (range)	73.5 ± 5.5 (65–82)	71.7 ± 6.4 (65–86)	72.6 ± 6.0 (65–86)	0.44
Living situation, n (%)				
Alone	5 (38%)	7 (47%)	12 (43%)	0.72
With spouse	8 (62%)	8 (53%)	16 (57%)	
Currently or previously self-isolated because of COVID-19 restrictions, n (%)				
Currently	8 (62%)	14 (93%)	22 (79%)	
Previously	5 (38%)	1 (7%)	6 (21%)	0.07
Currently or previously quarantined because of COVID-19 restrictions, n (%)				
Currently	0 (0%)	2 (13%)	2 (7%)	
Previously	3 (23%)	3 (20%)	7 (25%)	
Never	10 (77%)	10 (67%)	19 (68%)	0.39
Annual income, CAD $ ± SD	94,367 ± 89,592	48,387 ± 27,783	69,734 ± 67,179	0.08
Financial screen—Yes, n (%)	--	--	3 (11%)	
Food insecurity screen—Yes, n (%)	--	--		
Resilience ± SD (score)	3.9 ± 0.5	3.3 ± 1.0	1 (4%)	0.06
Nutrition risk ± SD (score)	44.9 ± 8.9	44.2 ± 10.3	44.6 ± 9.5	0.85

Normal resilience range measured using the BRS is 3.6–4.3. Nutrition risk was scored as low risk (≥50) or high risk (<50) with SCREEN-14. Fisher’s Exact Test was used to test for significance of categorical variables and unpaired *t*-test for continuous variables.

**Table 2:** Change in Nutrition Risk, Awareness and Usage of Food-related Services and Resources.

		Nutrition Risk			*p*-Value		
		Baseline		Post			
Nutrition Risk Score	SCREEN-14 Score (Mean ± SD)	44.6 ± 9.5		44.6 ± 9.7	0.96		
		**Awareness of** **S and R**		***p*-Value**	**Use of** **S and R**		***p*-Value**
		**Baseline**	**Post**		**Baseline**	**Post**	
Services and Resources *	Composite score (mean ± SD)	10 ± 4	11 ± 4	0.01	2 ± 2	3 ± 2	0.57
		**N (%)**	**N (%)**		**N (%)**	**N (%)**	
*Transportation or Food Delivery*	*All (mean of 5 S and R)*	*11 (39)*	*21 (75)*	*0.01*	*4 (14)*	*3 (11)*	1.0
	Transportation services	16 (57)	18 (64)	0.79	4 (14)	3 (11)	1.0
	Restaurant or fast-food delivery	28 (100)	28 (100)	1.0	8 (29)	8 (29)	1.0
	Online grocery shopping and/or delivery	27 (96)	28 (100)	1.0	6 (21)	7 (25)	1.0
	Meals on Wheels^TM^	26 (93)	28 (100)	0.49	0 (0)	0 (0)	1.0
	Bag Half Full^TM^	1 (4)	2 (7)	1.0	0 (0)	0 (0)	1.0
*Telephone Services*	*All (mean of 3 S and R)*	*15 (55)*	*17 (62)*	*0.79*	*5 (17)*	*3 (11)*	0.71
	Alberta 211 Helpline	17 (61)	17 (61)	1.0	2 (7)	2 (7)	1.0
	Health Link 811	27 (96)	27 (96)	1.0	12 (28)	8 (29)	0.40
	Rehabilitation Advice Line	2 (7)	4 (14)	0.67	0 (0)	0 (0)	1.0
*Websites*	*All (mean of 4 S and R)*	*7 (25)*	*9 (32)*	*0.79*	*4 (14)*	*4 (14)*	1.0
	Healthy Aging CORE^TM^	2 (7)	4 (14)	0.67	0 (0)	1 (4)	1.0
	MyHealth Alberta	15 (54)	17 (61)	0.79	11 (39)	11 (39)	1.0
	AHS “Healthy Eating Starts Here”	9 (32)	11 (39)	0.78	3 (11)	4 (14)	1.0
	Communities Choosewell^TM^	1 (4)	3 (11)	0.61	0 (0)	0 (0)	1.0
*Nutrition Counselling*	*All (mean of 5 S and R)*	*13 (46)*	*16 (57)*	*0.59*	*3 (11)*	*5 (18)*	*0.71*
	Family doctor	18 (64)	19 (68)	1.0	7 (25)	7 (25)	1.0
	AHS nutrition counselling	13 (46)	17 (61)	0.42	3 (11)	6 (21)	0.47
	City of Edmonton nutrition services	3 (11)	8 (29)	0.18	0 (0)	0 (0)	1.0
	Dietitians of Canada or other online directories to find a Registered Dietitian	13 (46)	17 (61)	0.42	3 (11)	4 (14)	1.0
	Primary care network-based nutrition counselling	17 (61)	17 (61)	1.0	3 (11)	7 (25)	0.30
*Food Security*	*All (mean of 3 S and R)*	*14 (50)*	*17 (61)*	*0.59*	*<1 (1)*	*1 (4)*	*1.0*
	Food banks/food hampers	24 (86)	25 (89)	1.0	0 (0)	0 (0)	1.0
	Community groups providing low-cost prepared or frozen meals	17 (61)	21 (75)	0.39	1 (4)	2 (7)	1.0
	Free Food in Alberta directory	1 (4)	6 (21)	0.10	0 (0)	1 (4)	1.0

* Composite score out of 20 total S and R, compared by unpaired *t*-test. Categorical data (answered “yes” to aware of and/or use of S and R) compared by Fisher’s exact test. The total S and R score is highlighted in bold as are major headers within the table. Major sub-categories of S and R are shown in italics with individual S and R in each sub-category shown in plain text.

**Table 3:** Individual SCREEN-14 Items at Follow-Up Stratified by High and Low Nutrition Risk.

Nutrition Risk Factor	Education Provided in Handouts	High Risk (N = 21)	Low Risk (N = 7)	*p*-Value
Inadequate servings of milk, milk products, soy	Yes	1.2 ± 1.2	2.6±1.0	0.02
Perception that weight is more/less than it should be	Yes	1.3 ± 0.19	1.1 ± 2.0	0.82
Inadequate servings of fruits and vegetables	Yes	1.9 ± 1.3	3.7 ± 0.5	<0.01
Eating alone	No	1.9 ± 1.8	2.9 ± 2.0	0.24
Limiting or avoiding foods	No	2.0 ± 1.3	2.9 ± 1.1	0.12
Inadequate protein intake	Yes	2.0 ± 1.3	3.6 ± 0.5	<0.01
Weight gain/loss ≥ 5 l b	Yes	2.6 ± 1.6	3.1 ± 1.5	0.41
Skipping meals	Yes	2.7 ± 1.5	4.0 ± 0	0.04
Inadequate fluid intake	Yes	2.9 ± 0.8	3.7 ± 0.5	0.02
Difficulty with meal preparation	*	3.0 ± 1.5	3.4 ± 1.0	0.44
Coughing, choking or pain swallowing when eating	*	3.1 ± 1.3	3.9 ± 0.4	0.16
Unintentional weight gain/loss	Yes	3.4 ± 1.4	4.0 ± 0	0.31
Poor appetite	*	3.5 ± 1.0	3.0 ± 0.8	0.23
Difficulty chewing	*	3.5 ± 1.0	4.0 ± 0	0.22
Problems obtaining groceries	*#	3.5 ± 1.0	4.0 ± 0	0.24
Uses commercial meal replacements or supplements	Yes	3.9 ± 0.9	3.7 ± 0.8	0.47

Means ± SD for individual SCREEN-14 items sorted from lowest score (highest risk) to highest score (lowest risk). Scores of ≤2 (out of 4) on individual SCREEN-14 questions help identify the specific behavior or factor contributing to nutrition risk. Differences between low- and high-risk groups were compared using unpaired *t*-test. * Limited information in the AHS handout(s) but information on where to get more help was provided. # Information provided in the S and R.

## Data Availability

Data are available upon reasonable request to the corresponding author.
